# Delta neutrophil index, CRP/albumin ratio, procalcitonin, immature granulocytes, and HALP score in acute appendicitis: Best performing biomarker?

**DOI:** 10.1515/med-2025-1308

**Published:** 2025-10-16

**Authors:** Mustafa Oguz Cumaoglu, Durmus Ayan, Abdussamed Vural, Turgut Dolanbay, Caner Ozbey, Abdul Ridvan Kulu

**Affiliations:** Department of Emergency Medicine, Nigde Omer Halisdemir University Faculty of Medicine, Nigde, Turkey; Department of Emergency Medicine, Malatya Turgut Ozal University School of Medicine, Malatya, Turkey; Department of Pathology, Nigde Education and Research Hospital, Nigde, Turkey; Department of Medical Biochemistry, Nigde Omer Halisdemir University Faculty of Medicine, Nigde, Turkey; Department of Surgery, Nigde Omer Halisdemir University Faculty of Medicine, Nigde, Turkey

**Keywords:** acute appendicitis, delta neutrophil index, diagnosis, emergency department, inflammatory biomarker

## Abstract

**Aim:**

This study investigates the diagnostic accuracy of five inflammatory biomarkers – delta neutrophil index (DNI); C-reactive protein/albumin ratio (CAR); procalcitonin (PCT); immature granulocytes count (IGc); and hemoglobin, albumin, lymphocyte, platelet score (HALPs) – in detecting acute appendicitis (AA) and identifying the most reliable marker for distinguishing complicated from non-complicated cases.

**Materials and methods:**

A prospective, cross-sectional study was conducted in a tertiary hospital emergency department, including 100 histopathologically confirmed AA patients and 100 healthy controls. Biomarker levels were analyzed using receiver operating characteristic curves. Sensitivity, specificity, and optimal cut-off values were determined via the Youden index, while logistic regression identified independent predictors of complicated appendicitis (CA).

**Results:**

DNI exhibited the highest diagnostic accuracy for AA (AUC: 0.87, 95% CI: 0.821–0.919, *p* < 0.001) with 92% specificity and 69% sensitivity at a cut-off of 71.88. CAR (AUC: 0.853) and PCT (AUC: 0.852) showed similar performance, whereas IGc (AUC: 0.695) and HALPs (AUC: 0.627) were less effective. DNI also outperformed the Alvarado score in predicting CA (AUC: 0.698 vs 0.626) and was an independent predictor (OR: 3.18, 95% CI: 1.40–7.24, *p* = 0.006).

**Conclusions:**

DNI demonstrated superior diagnostic performance for AA and was the most reliable marker for identifying CA. Its integration into clinical practice may enhance early diagnosis and reduce unnecessary surgeries, improving outcomes.

## 1 Introduction

Acute appendicitis (AA) is a surgical emergency characterized by obstruction of the vermiform appendix due to fecalith, lymphoid hyperplasia, or foreign bodies, leading to increased intraluminal pressure, impaired venous flow, and subsequent bacterial infection [1]. Its annual incidence is approximately 90–100 per 100,000 individuals, making it one of the most common causes of acute abdominal pain, accounting for 7–8% of all emergency department (ED) visits [2]. In the early stages, the inflammatory process remains confined to the appendix itself, but as the condition progresses, it can lead to life-threatening complications such as peritonitis, necrosis, and perforation. Therefore, early and accurate diagnosis is crucial for preventing complications and reducing unnecessary surgical interventions [3].

The diagnostic process for AA involves a combination of clinical evaluation, laboratory tests, and imaging modalities such as ultrasonography or contrast-enhanced computed tomography (CT) scans [4]. Although imaging techniques have high sensitivity and specificity, their limitations, including high cost, limited accessibility, and radiation exposure, have increased interest in biochemical marker-based diagnostic approaches in emergency settings [5]. AA may present with atypical symptoms in the early stages, leading to diagnostic uncertainty and potential mismanagement. Thus, identifying more reliable and rapid diagnostic biomarkers holds significant clinical value [6]. In recent years, studies have explored the diagnostic value of novel inflammatory biomarkers, including delta neutrophil index (DNI); C-reactive protein/albumin ratio (CAR); procalcitonin (PCT); immature granulocyte count (IGc); and hemoglobin, albumin, lymphocyte, platelet score (HALPs), beyond traditional markers such as leukocytes and neutrophils. Each of these biomarkers provides insight into different stages of the inflammatory cascade [7–11]. A literature review revealed that most studies investigating these five biomarkers in AA were designed retrospectively. However, no comprehensive prospective study has evaluated their diagnostic accuracy as a whole or compared their predictive performance systematically.

To address this gap, the present study aims to provide a broad-spectrum analysis of these five inflammatory biomarkers, assess their sensitivity and specificity in predicting AA, and determine the most reliable biomarker for early and accurate diagnosis in clinical practice.

## 2 Materials and methods

### 2.1 Study design

This single-center, prospective, cross-sectional diagnostic accuracy study was conducted in the ED of a tertiary university hospital, which evaluates approximately 520,000 patients annually. Patient enrollment began after obtaining approval from the institutional ethics committee. The study was conducted between February 9, 2024 and September 19, 2024. Throughout the study, all procedures adhered to the ethical principles outlined in the Declaration of Helsinki.

The patient group consisted of individuals who presented to the ED with clinical, laboratory, and imaging findings suggestive of AA and subsequently underwent appendectomy. Additionally, the diagnosis of AA was confirmed postoperatively through histopathological examination. The control group comprised healthy volunteers who met the inclusion criteria and exhibited no clinical symptoms related to AA. These individuals were selected to ensure no statistically significant differences in age and sex compared to the patient group. Both groups were provided with detailed information about the study, and written informed consent was obtained from all participants.

### 2.2 Study population

The study population consisted of two main groups: patients with AA and healthy individuals. The AA group was further subdivided into complicated (CA) and non-complicated (NCA) cases based on histopathological findings.

The inclusion and exclusion criteria for the patient and control groups were as follows: individuals younger than 18 years, pregnant women, those with a history of surgery within the past 6 months, patients with diabetes, acute liver failure, acute renal failure, hematologic disorders, malignancy or metastasis, immunodeficiency, or a history of immunosuppressive drug use were excluded. Additionally, individuals who had used antibiotics within the week prior to admission or withdrew their consent during the study were not included.

### 2.3 Study protocol

Patients presenting to the ED with abdominal pain that had started within the past 24 h and exhibiting clinical and physical examination findings consistent with AA (localized right lower quadrant pain, guarding, rebound tenderness, nausea, vomiting, etc.) were informed about the study. Blood samples were collected from patients who met the inclusion criteria and agreed to participate. Hematological parameters were measured using the Sysmex XN-1000 (Kobe, Japan) device with fluorescent flow cytometry. C-reactive protein (CRP) and albumin levels were analyzed via spectrophotometry using the Roche Cobas c701 (Kobe, Japan) system, while serum PCT levels (ng/mL) were determined via electrochemiluminescence using the Roche Cobas E801 (Kobe, Japan) device. The coefficient of variation and quality control data for hemogram parameters, as well as the intra-laboratory repeatability and manufacturer-reported precision data for CRP, albumin, and PCT tests, are presented in Supplementary materials 1 and 2.

The following inflammatory biomarkers were calculated: DNI [(%neutrophil + %eosinophil) − (%polymorphoneutrophil (IG)], CRP (mg/L)/albumin (g/L) ratio [CAR], IGc (µL), and HALPs [(hemoglobin (g/L) × albumin (g/L) × lymphocyte (10^3^/mcL))/platelet (10^3^/mcL)]. Data collection forms recorded demographic variables such as age and sex, vital signs, and Alvarado score calculations. The Alvarado scoring system is presented in Supplementary material 3 [12].

Patients scoring ≥5 on the Alvarado scale were classified as medium-to-high risk for AA and underwent radiological evaluation, including ultrasonography and/or contrast-enhanced abdominal CT, provided they had no contraindications (e.g., history of contrast allergy, abnormal renal function). Patients with radiologic findings suggestive of appendicitis (e.g., anteroposterior appendix diameter ≥6 mm, absence of luminal air, etc.) were admitted to the surgical ward following evaluation by an experienced general surgeon. Patients with scores <5 were assessed jointly by an emergency medicine specialist and a general surgeon; those with persistent suspicion of AA were admitted for further observation, additional testing, and treatment.

Following hospitalization, appendectomy specimens were fixed in formalin, embedded in paraffin, sectioned at 4 µm thickness, stained with hematoxylin–eosin, and examined under a light microscope. Patients with inflammation confined to the appendix tissue were classified as NCA, whereas those exhibiting periapendiceal fat inflammation, peritonitis, necrosis, gangrenous appendicitis, or perforation were classified as having CA. Anatomical localization of the appendix (e.g., retrocecal, pelvic, subcecal) was also recorded intraoperatively.

The control group consisted of healthy individuals who met the inclusion criteria and voluntarily participated. Blood samples were collected from control subjects, and the same biomarker measurements and calculations were performed. The biomarker levels were compared between the AA and control groups, as well as between the CA and NCA subgroups. Furthermore, Alvarado scores were analyzed alongside biomarker levels to assess their predictive accuracy in diagnosing CA.

### 2.4 Outcome measures

The primary outcome of the study was to identify the biomarker among these five with the highest sensitivity and specificity in predicting AA. By doing so, a single biomarker would stand out, providing researchers with a valuable dataset for future studies. The secondary outcome was to determine the biomarker that demonstrated superior predictive value for CA in subgroup analyses.

### 2.5 Data analysis

Statistical analyses were performed using the Jamovi software package (Jamovi Project, version 1.6, https://www.jamovi.org) and SPSS v.27 (IBM Corp., Armonk, NY, USA). Normality of data distribution was assessed using both visual (histogram, stem-and-leaf plots, scatter plots, and box plots) and analytical methods (Kolmogorov–Smirnov test, skewness, and kurtosis). Continuous variables were analyzed using the Mann–Whitney *U*-test, and results were reported as median and interquartile range (IQR: 25–75). Categorical variables were analyzed using the chi-square test or Fisher’s exact test, with results presented as frequencies and percentages.

To evaluate the diagnostic and subgroup performance of DNI, CAR, PCT, IGc, and HALPs in predicting AA, as well as the Alvarado score in subgroup analyses, a receiver operating characteristic (ROC) curve analysis was conducted. Cut-off values in the ROC curve analysis were determined using the Youden index. Sensitivity, specificity, positive likelihood ratio (+LR), and negative likelihood ratio (−LR) were calculated for the optimal cut-off points of these parameters. Patients with appendicitis were categorized into two groups (low and high) based on the median value of their DNI results. The potential role of DNI as an independent predictor of CA was assessed using binary logistic regression analysis. Odds ratio value was calculated, and differences were reported with 95% confidence intervals (CIs). A *p*-value of <0.05 was considered statistically significant (Type 1 error threshold).

### 2.6 Sample size

Since there are no similar studies comparing five different inflammation-related biomarkers in predicting appendicitis diagnosis, a sample size of 140 individuals was determined to be necessary to achieve 80% power with an effect size of 0.5, considering a 10% margin of error. This calculation was performed using the G-Power 3.1.9.7 software (Heinrich Heine Universität, Düsseldorf). The total sample size of this study consists of 200 individuals.


**Informed consent:** The informed consent form was read and explained to both the patient and the control group. After obtaining their signed consent forms, they were included in the study.
**Ethical approval:** In order to carry out this research, approval was obtained from the non-interventional ethics committee Nigde Omer Halisdemir University with the letter numbered No.: 2024/14, Date: 08.02.2024.

## 3 Results

A total of 200 individuals aged between 18 and 77 years were included in the study, comprising 100 patients with AA and 100 control subjects. The median age of the AA group was 35 years (IQR: 25.25–50), while that of the control group was 31 years (IQR: 25–40). Males accounted for 58% of the AA group and 70% of the control group.

In addition to the five targeted inflammatory biomarkers, routine emergency laboratory parameters – including leukocyte, hemoglobin, platelet, glucose, urea, creatinine, alanine aminotransferase, and aspartate aminotransferase – were also compared between the appendicitis and control groups. These results are presented in Supplementary material 4.

In the AA group, DNI, CAR, PCT, and IGc levels were significantly higher compared to the control group, whereas the HALPs was significantly lower (*p* < 0.05). In the appendicitis subgroup analysis, 54 patients had NCA, while 46 had CA. The CA group exhibited significantly higher DNI and Alvarado scores (*p* < 0.05). However, there were no significant differences between the two subgroups in terms of CAR, PCT, IGc, or HALPs (*p* > 0.05) (Table 1). Appendix positions included pelvic (33%), retrocecal (23%), paraileal (23%), and paracecal (21%). However, no statistically significant differences were observed between anatomical subgroups in terms of the five evaluated biomarkers (*p* > 0.05 for all).

**Table 1:** Comparison of biomarkers according to control-AA groups and appendicitis subgroups

	Control (*n* = 100)	AA (*n* = 100)	*p* value	NCA (*n* = 54)	CA (*n* = 46)	*p* value
DNI	59.48 (54.69–64.32)	77.30 (66.97–83.44)	**<0.001**	74.54 (64.16–80.95)	81.59 (72.20–88.60)	**<0.001**
CAR	0.021 (0.013–0.054)	0.32 (0.07–1.6)	**<0.001**	0.20 (0.02–1.12)	0.36 (0.13–1.73)	0.075
PCT	0.03 (0.02–0.05)	0.083 (0.052–0.229)	**<0.001**	0.07 (0.03–0.23)	0.08 (0.05–0.22)	0.226
IGc	0.01 (0.01–0.02)	0.03 (0.01–0.04)	**<0.001**	0.02 (0.01–0.03)	0.03 (0.02–0.04)	0.089
HALPs	6.11 ( 4.64–7.76)	4.81 (3.04–6.80)	**0.002**	4.85 (3.19–6.84)	4.54 (2.6–6.78)	0.623
Alvarado score				6 (5–8)	8 (7–8)	**0.027**

DNI: delta neutrophil index, CAR: C-reactive protein/albumin ratio, PCT: procalcitonin, IGc: immature granulocyte count, HALP: hemoglobin albumin lymphocyte platelet score, IQR: interquartile range, AA: acute appendicitis, CA: complicated appendicitis, NCA: non-complicated appendicitis; biomarkers and the Alvarado score are presented as median (IQR: 25–75). Bold values denote statistically significant results (p < 0.05).

**Figure 1:**
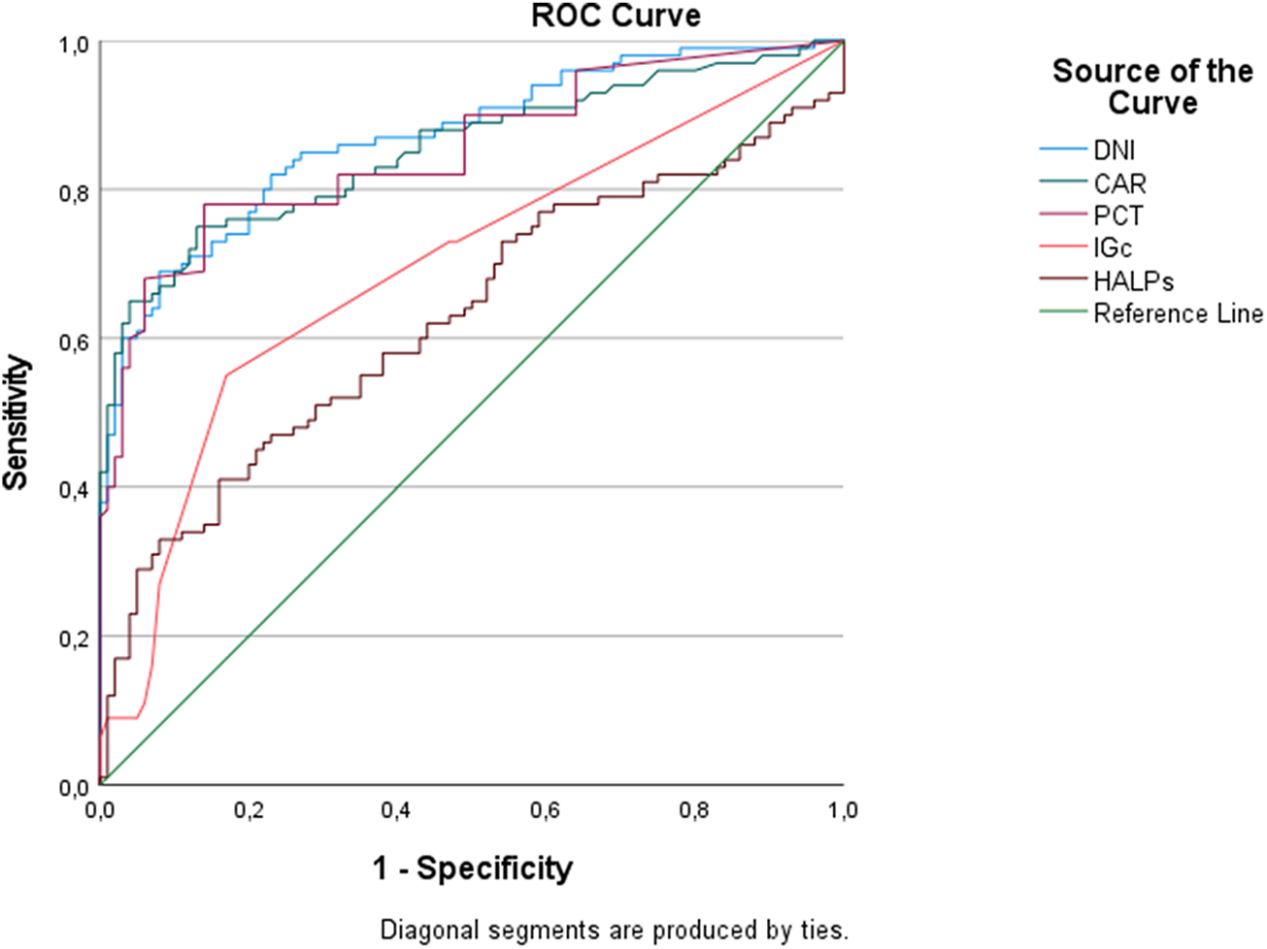
Predicting acute appendicitis.

**Figure 2:**
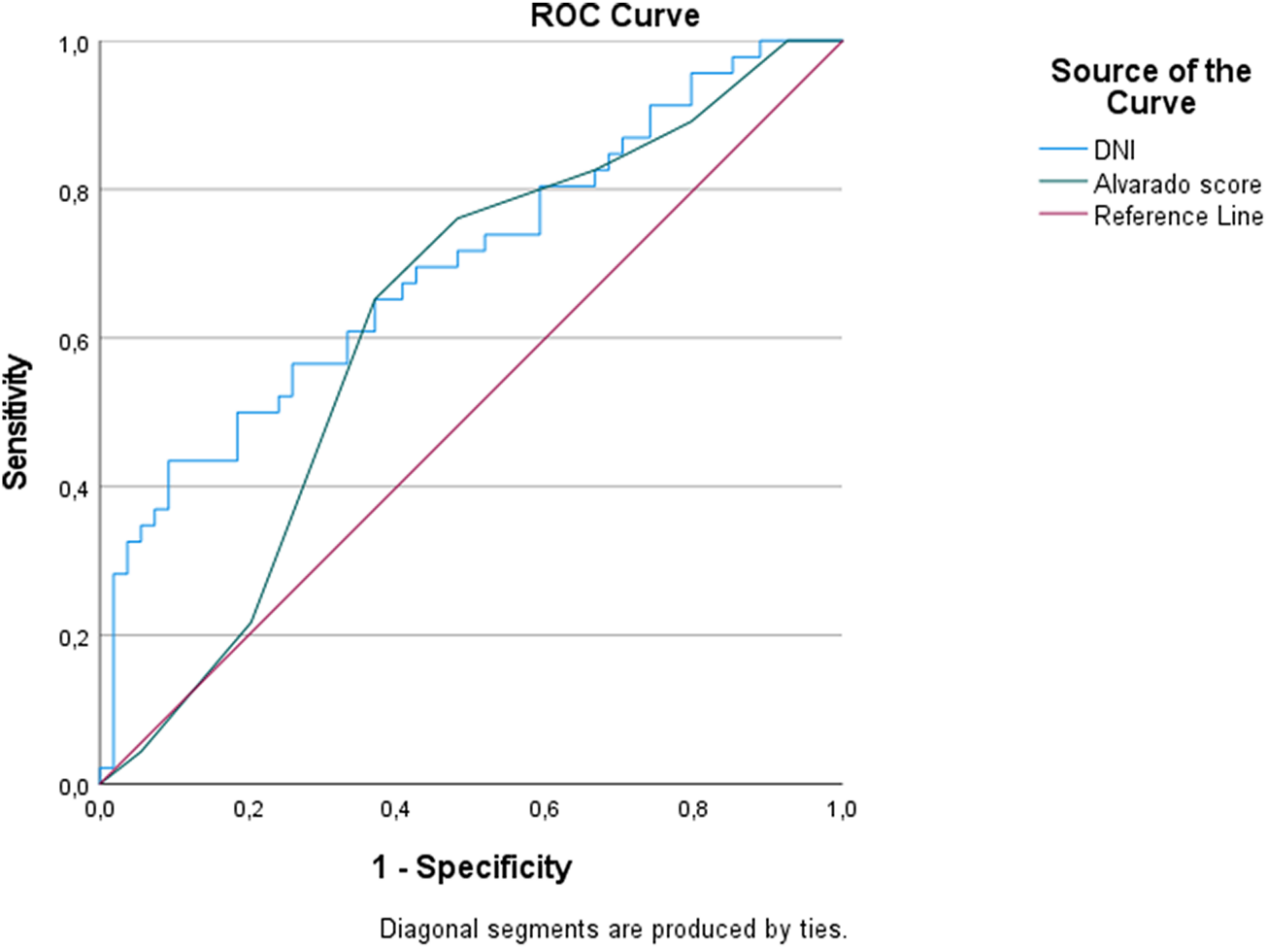
Predicting complicated appendicitis.

According to ROC analysis results; DNI demonstrated the highest diagnostic accuracy in predicting AA. At a cut-off value of 71.88, DNI had a specificity of 92% and a sensitivity of 69% for AA. CAR and PCT exhibited similar performance. However, IGc and the HALPs demonstrated lower diagnostic accuracy. In predicting CA, DNI showed a higher diagnostic performance than the Alvarado score. At a cut-off value of 83.44, DNI had a specificity of 90% and a sensitivity of 43% for CA (Table 2, Figures 1 and 2).

**Table 2:** ROC analysis results

		AUC	95% CI	Youden index	Cut-off	Sensitivity	Specificity	+LR	−LR	*p* value
Control-AA groups										
	DNI	0.870	0.821–0.919	0.61	71.88	0.69	0.92	8.62	0.33	<0.01
	CAR	0.853	0.800–0.907	0.62	0.081	0.75	0.87	5.76	0.28	<0.01
	PCT	0.852	0.799–0.905	0.64	0.05	0.78	0.86	5.57	0.25	<0.01
	IGc	0.695	0.621–0.769	0.38	0.025	0.55	0.83	3.23	0.54	<0.01
	HALPs	0.627	0.549–0.706	0.25	0.246	0.41	0.84	2.56	0.70	0.02
NCA-CA										
	DNI	0.698	0.590–0.800	0.342	83.44	0.43	0.90	4.67	0.62	<0.001
	Alvarado score	0.626	0.510–0.730	0.282	7.5	0.65	0.63	1.76	0.55	0.03

ROC: receiver operating characteristic, AUC: area under the curve, CI: confidence interval, LR: likelihood ratio, AA: acute appendicitis, CA: complicated appendicitis, NCA: non-complicated appendicitis, DNI: delta neutrophil index, CAR: C-reactive protein/albumin ratio, PCT: procalcitonin, IGc: immature granulocyte count, HALPs: hemoglobin albumin lymphocyte platelet score.

Binary logistic regression analysis demonstrated that DNI was an independent predictor of CA. The likelihood of predicting CA significantly increased at DNI values of ≥77.3 (OR: 3.18, 95% CI: 1.40–7.24, *p* = 0.006).

## 4 Discussion

To the best of our knowledge, this is the first prospective diagnostic accuracy study evaluating DNI, CAR, PCT, IGc, and HALPs collectively in the diagnostic process of AA in the ED and in appendicitis subgroup analysis. Among these biomarkers, DNI demonstrated the best performance in diagnosing AA. While CAR and PCT exhibited good performance with similar AUC values, IGc and HALPs showed moderate diagnostic accuracy. In predicting CA, only DNI was identified as an independent predictor at a clinically usable level.

The fundamental pathophysiology of appendicitis is based on the local and systemic inflammatory response triggered by the immune system following mechanical obstruction [13]. Neutrophils are the first immune cells to reach the site of inflammation, and in cases of AA, neutrophil-dominant infiltration is observed in the muscularis propria layer [14]. It has also been shown that before neutrophil infiltration, an eosinophilic infiltration occurs in the muscularis propria during the early inflammatory phase [15]. DNI is an indicator of immature neutrophils, calculated by subtracting the percentage of polymorphonuclear neutrophils from the total percentage of circulating neutrophils and eosinophils. The elevated DNI levels observed in AA can be explained by the increased activity of myeloid progenitor cells during the acute inflammatory response [7,16]. Previous studies support the utility of DNI in diagnosing AA. Shin et al. [17] reported that DNI is a valuable parameter for predicting both AA and CA among patients who underwent surgical intervention. Similarly, Dinc et al. [18], in a retrospective analysis, classified patients into simple and CA groups based on post-appendectomy histopathological findings and demonstrated that DNI is a significant marker for predicting CA. Our findings positively align with the existing literature regarding DNI. It outperformed the other four biomarkers in both the diagnostic process of AA and in predicting CA.

During the acute inflammatory phase of appendicitis, the positive acute-phase protein CRP increases, while the negative acute-phase protein albumin decreases. In the presence of high inflammation, the CAR becomes a more specific and reliable marker compared to using these two inflammatory predictors individually [19]. Akbas et al. [20] reported that CAR is a useful indicator for diagnosing AA in pregnant patients, a group in which the diagnostic and surgical decision-making process is particularly challenging. Some studies have also suggested that CAR may serve as a predictor of CA [19,21]. However, in this study, while CAR demonstrated relatively high performance in diagnosing AA, it did not yield the expected results in the appendicitis subgroup analysis.

PCT is a precursor of the hormone calcitonin, produced by thyroid parafollicular cells. Its levels increase in bacterial infections due to the effects of endotoxins, lipopolysaccharides, and inflammatory mediators, while they decrease in viral infections [22]. Most studies in the literature have investigated PCT in distinguishing CA from NCA and analyzing the subgroups of CA. These studies have reported significantly elevated PCT levels, particularly in the presence of gangrenous, necrotic, and perforated appendicitis [9]. However, Motie et al. [23] recommended against the use of PCT in the diagnostic process of AA. In contrast to previous literature, we identified PCT as an important biomarker for diagnosing AA, demonstrating a similar AUC level to CAR. Although PCT levels were higher in patients with CA compared to NCA cases, this difference was not statistically significant.

Immature granulocytes are premature granulocytes (promyelocytes, myelocytes, metamyelocytes) released into the bloodstream from the bone marrow in response to inflammation. They are neutrophil precursors that emerge earlier than neutrophil band forms. With the advancement of next-generation hematology analyzers, IGc can now be easily measured through a complete blood count test [24]. There are only a few studies investigating the relationship between IGc and AA [10,25]. These studies have suggested that IGc is a significant marker for predicting both AA and CA but emphasized the need for prospective studies to confirm these findings. According to our data, IGc was useful only in the diagnostic process of AA. While it may aid in diagnosing AA, it is not a reliable marker for distinguishing CA and should not be used alone for this purpose.

The HALP score is a novel marker formulated through the combination of hemoglobin, albumin, lymphocytes, and platelets, allowing for an assessment of patients’ systemic inflammation and nutritional status. In the presence of a low HALP score, negative interpretations can be made regarding the severity and complication status of AA [11]. Saridas et al. [26] stated that the HALP score lags behind inflammation-related markers such as the pan-immune inflammation value and the systemic immune inflammation index in predicting CA. According to this study, the HALP score can be used to predict AA; however, it should be noted that it exhibits the lowest performance compared to other markers. It is ineffective in predicting CA.

Despite the promising performance of DNI in predicting both AA and CA, one important limitation must be acknowledged. In our analysis, the sensitivity of DNI in identifying CA was found to be relatively low (43%) at the optimal cut-off value (83.44). This limited sensitivity suggests that a significant proportion of patients with CA might not be detected using this marker alone, potentially leading to underdiagnosis and delayed surgical intervention. While DNI offers high specificity (90%) and thus minimizes false-positive results, its insufficient sensitivity underscores the necessity of combining it with clinical evaluation and other diagnostic tools in practice. Therefore, DNI should not be used in isolation for ruling out CA, but rather as a complementary parameter that can enhance diagnostic confidence when interpreted within the broader clinical context.

### 4.1 Study limitations

The study has certain limitations, such as being a single-center study and the relatively small sample size in the subgroup analysis of CA and NCA. In the future, conducting multicenter studies with larger sample sizes will be important for validating these findings and further refining the role of inflammatory biomarkers in the diagnosis and classification of AA. Additionally, the relatively low sensitivity of DNI in predicting CA may restrict its standalone diagnostic utility and should be interpreted cautiously in clinical settings.

While the control group was composed of healthy individuals – a standard approach in biomarker accuracy studies – we acknowledge that this may not fully reflect the complex diagnostic landscape of the ED. This should be taken into account when interpreting the clinical applicability of our findings. Future studies comparing appendicitis patients with other common mimicking conditions may help validate and refine our findings.

## 5 Conclusion

This prospective cross-sectional study provides a comprehensive evaluation of five different inflammatory biomarkers (DNI, CAR, PCT, IGc, and HALPs) in the diagnosis and subgroup analysis of AA. Among these biomarkers, DNI demonstrated the highest diagnostic accuracy, making it the most reliable parameter for predicting AA. Furthermore, DNI outperformed other biomarkers and even the Alvarado scoring system in diagnostic performance, establishing itself as an independent marker for CA. This study highlights the clinical value of DNI, suggesting that it can contribute to the early and accurate diagnosis of AA and help reduce unnecessary surgical interventions.

## Supplementary Material

Supplementary Table
