# Supplementary material for: Delta neutrophil index, CRP/albumin ratio, procalcitonin, immature granulocytes, and HALP score in acute appendicitis: Best performing biomarker?
Source: Open Med (Wars). 2025 Oct 16;20(1):20251308. doi: 10.1515/med-2025-1308 (PMC12552854; doi:10.1515/med-2025-1308)
Supplement: Supplementary Table [file med-2025-1308-sm.pdf]

Supplementary material

Table S1: CV and QC data of hemogram parameters

	QC Level 1	QC Level 3	QC Level 1	QC Level 3	%CV
Neutrophils (%)	5.25	1.28	2.85	2.11	<8.0
Eosinophils (%)	8.45	8.18	6.94	6.62	<25.0
Immature granulocyte (%)	6.10	2.67	3.43	2.42	<25.0
Immature granulocyte (×10^3/μL)	6.24	2.24	4.33	2.56	<25.0
Hemoglobin (g/L)	0.96	0.37	0.93	0.88	<1.0
Lymphocytes (×10^3/mcL)	1.20	3.09	5.75	2.78	<8.0
Platelet (×10^3/mcL)	2.66	2.22	3.68	2.21	<4.0
Leucocyte (×10^3/μL)	1.57	2.25	1.92	2.16	<3.0
Neutrophils (×10^3/mcL)	4.56	1.58	3.14	2.29	<8.0

CV: Coefficient of variation, QC: Quality control.

Table S2: Repeatability and precision measurements for CRP, albumin, and PCT

	Intra-laboratory repeatability measurements			Manufacturer-reported precision values		
	**%CV 1**	**%CV 2**	**%CV 3**	**%CV 1**	**%CV 2**	**%CV 3**
CRP	2.5 (5.1 ± 0.13)	2.3 (351 ± 7.99)		3.3 (5.1 ± 0.17)	2.4 (351 ± 2.4)	
Albumin	0.7 (51.3 ± 0.4)	1.2 (42.4 ± 0.5)		0.9 (51.3 ± 0.5)	1.0 (42.2 ± 0.4)	
PCT	7.1 (0.08 ± 0.007)	1.8 (0.431 ± 0.008)	1.1 (54.4 ± 0.618)	8.7 (0.08 ± 0.007)	2.6 (0.431 ± 0.011)	1.6 (54.4 ± 0.895)

CRP: C-reactive protein, PCT: Procalcitonin, CV: Coefficient of variation.

Table S3: Alvarado score for acute appendicitis

Right lower quadrant tenderness	2 points
Elevated temperature (37.3°C or 99.1°F)	1 point
Rebound tenderness	1 point
Migration of pain to the right lower quadrant	1 point
Anorexia	1 point
Nausea or vomiting	1 point
Leukocytosis >10,000	2 points
Leukocyte left shift (>75% neutrophils)	1 point
<4 points: Low probability of appendicitis, no need for additional imaging.	
5-6 points: Acute appendicitis is possible.	
7-8 points: Acute appendicitis is likely.	
9-10 points: Highly likely acute appendicitis.	

Table S4: Comparison of routine laboratory parameters between groups

Parameters	Control (n = 100)	Appendicitis (n = 100)	p value
Leukocyte, $\times 10^9$/L	7.13 (5.93 to 8.67)	12.5 (9.22 to 18.57)	<**0.001**
Hemoglobin, g/dL	15.55 (14 to 16.17)	14.65 (13.12 to 15.87)	**0.018**
Platelet, $\times 10^9$/L	267.5 (232.25 to 309.75)	266.5 (221.25 to 312.75)	0.738
Glucose, mg/dL	84.31 ± 6.27	86.2 ± 7.05	**0.047**
Urea, mg/dL	14.17 ± 2.85	13.72 ± 3.05	0.277
Creatinine, mg/dL	1 (0.85 to 1.11)	1,03 (0.85 to 1.17)	0.197
ALT, U/L	30.72 ± 9.92	29.99 ± 11.07	0.622
AST, U/L	24.17 ± 5.61	25.26 ± 6.39	0.200

Data are presented as median (Interquartile range: 25 to 75) or mean ± standard deviation.

ALT: Alanine aminotransferase, AST: Aspartate aminotransferase.
